# Patterns and determinants of dropout from maternity care continuum in Nigeria

**DOI:** 10.1186/s12884-016-1083-9

**Published:** 2016-09-27

**Authors:** Joshua O. Akinyemi, Rotimi F. Afolabi, Olutosin A. Awolude

**Affiliations:** College of Medicine, University of Ibadan, Ibadan, Nigeria

**Keywords:** Maternity care continuum, Dropout, Education, Poverty, Healthcare access problems, Nigeria

## Abstract

**Background:**

The maternal, newborn and child health care continuum require that mother/child pair should receive the full package of antenatal, intrapartum and postnatal care in order to derive maximum benefits. Continuity of care is a challenge in sub-Saharan Africa. In this study, we investigate the patterns and factors associated with dropout in the continuum of maternity (antenatal, delivery and postnatal) care in Nigeria.

**Method:**

Using women recode file from the 2013 Nigeria Demographic and Health Survey, we analysed data on 20,467 women with an index birth within 5 years prior to data collection. Background characteristics and pattern of dropouts were summarised using descriptive statistics. The outcome variable was dropout which we explored in three stages: antenatal, antenatal-delivery, delivery-6 weeks postnatal visit. Multilevel logistic regression models were fitted to identify independent predictors of dropout at each stage. Measure of effect was expressed as Odds Ratio (OR) with 95 % confidence interval (CI).

**Results:**

Overall, 12,392 (60.6 %) of all women received antenatal care among whom 38.1 % dropout and never got skilled delivery assistance. Of those who received skilled delivery care, 50.8 % did not attend postnatal visit. The predictors of dropout between antenatal care and delivery include problem with getting money for treatment (OR = 1.18, CI: 1.04–1.34), distance to health facility (OR = 1.31, CI: 1.13–1.52), lack of formal education, being in poor wealth quintile (OR = 2.22, CI: 1.85–2.67), residing in rural areas (OR = 1.98, CI: 1.63–2.41). Regional differences between North East, North West and South West were significant. Between delivery and postnatal visit, the same factors were also associated with dropout.

**Conclusion:**

The rate of dropout from maternity care continuum is high in Nigeria and driven by low or lack of formal education, poverty and healthcare access problems (distance to facility and difficulty with getting money for treatment). Unexpectedly, dropouts are high in South east and South south as well as in the Northern regions. Intervention programs focusing on community outreach about the benefits of continuum of maternal healthcare package should be introduced especially for women in rural areas and lower socio-economic strata.

## Background

Despite differences in success stories across world regions, the millenium development goals (MDG) would have completed its course by end of 2015. Building on the MDG, the United Nations and countries of the world have adopted a new set of development agenda which has been captioned “Sustainable Development Goals (SDG)”. The SDG is proposed to comprise 17 goals unlike its predecessor which has 8. The third SDG is aimed at ensuring healthy lives and promote well being for all at all ages [1]. MDG 4 and 5 which was focused on child survival and maternal health respectively now constitute the first 2 targets under SDG 3 [1]. Meanwhile, the 2015 MDG report indicate that global under-5 mortality rate declined by more than half between 1990 and 2015 while maternal mortality ratio decreased by 45 % [2]. Since the MDG 4 and 5 could not be achieved in many parts of Sub-Saharan Africa, Latin Ameria and Oceania [1], it is justified to make them part of the post-2015 development agenda.

As part of efforts to fast track the progress in child survival and maternal health, the continuum of care for maternal, neonatal and child health was designed [3]. It includes healthcare services for mothers and children from pre-pregnancy to delivery, postnatal and childhood. This further underscore the inseparable link between maternal and child health as demonstrated by the fact that they are affected mostly by the same variables. The linkage between the 2 informed the design of packages for maternal, newborn and child health care (MNCH) continuum [4]. The continuum provides for healthcare packages over time and at different points with an overall aim of promoting maternal, newborn and child survival [5]. These specific time points are pre-pregancy, pregnancy, birth, newborn/postnatal and childhood. The idea of continuum require that mother/child pair should receive the full package in order to derive maximum benefits. Wenjuyan et al [6] provided evidence on the trends and factors affecting utilisation of maternal and child health services in developing countries by focusing on antenatal, delivery and postnatal care. While the likelihood of use of these services was associated with urban residence, higher education and rich/richer wealth quintiles, the trends however varied across countries.

The Nigeria Demographic Health Survey (NDHS) 2013 report indicated that out of all women aged 15–49 years who had a live birth in the 5 years before the survey, 61 % and 38 % received antenatal care (ANC) and skilled delivery assistance respectively [7]. This implied that nearly half of those who received antenatal dropped out before delivery. This default in continuity of care constitute missed opportunities and a risk factor for poor maternal and child health outcomes. The patterns and predictors of dropout in the MNCH care continuum need to be further investigated so that appropriate interventions can be suggested.

Most studies that investigated factors associated with maternal health service utilisation in Nigeria and other developing countries have treated antenatal, delivery and postnatal care as separate entities [6, 8, 9]. However, dropout in the MNCH care continuum have been investigated in Cambodia [10] and South Asia [11]. The Cambodia study showed that while 90 % received ANC, 71 % got skilled attendance at delivery and 60 % subsequently received postnatal care. That is 19 % and 11 % droped out before and after delivery respectively. A recent study on the continuum of care in 9 South Asia and sub-Saharan Africa countries revealed that more than half of women who commenced ANC did not attain up to 4 ANC visits [12]. The study further showed higher rates of completion of care continuum by women with higher education, rich wealth quintile and decision-making autonomy.

When women drop out of full range of MNCH care, they missed proper care that could enhance their own health and that of their newborn. At the population level, dropout reduces the impact of maternal and child health care on the overall level of morbidity and mortality among women and children. As efforts are made to improve the uptake of MNCH services, it is necessary to pay attention to cases of dropouts. Singh et al provided the most recent systematic investigation of the maternal care continuum in 6 sub-Saharan African countries (Ethiopia, Malawi, Rwanda, Senegal, Tanzania and Uganda) [12]. Though the findings provide very useful suggestions for regional programming, further country-specific studies will be necessary especially in Western and Central Africa where maternal and child health indicators are poorest.

In this study, we use data from Nigeria, the most populous country in the sub-region to investigate the pattern of dropout in the maternity (antenatal, delivery and postnatal) care continuum. The choice of country is apt considering its being one of the developing nations with slowest progress in maternal health improvement and child survival. Specifically, we addressed 2 questions: (1) what is the pattern of dropout from the maternity care continuum and (2) what are the factors associated with dropout.

## Methods

### Study setting

According to the World Bank, Nigeria is a lower-middle-income country with a population of 178.5 million people as at year 2014. There is wide geographical, ethnic, and health diversity as reflected in many of her demographic and health indices. Administratively, the country comprised of 36 states and a Federal Capital grouped into 6 geo-political zones: North West, North East, North Central, South East, South West and South- South. The population is young with 46 % being under 15 years. There are 3 tiers of government (Local, State and Federal) with each tier playing active roles in maternal and child health (MCH) care programmes. Total fertiility rate as at 2013 was 5.5 with 23 % of women aged 15–19 years having began childbearing [7]. The life expectancy at birth is 52 years. Under-5 mortality declined from 201 deaths per 1000 live births in 2003 to 128 per 1000 live births in 2013 while the maternal mortality ratio was 576 maternal deaths per 100, 000 live births [7]. There have been several programmes to promote MCH in the country but the introduction of the Midwives Service Scheme (MSS) in 2009 was a landmark initiative. The MSS was initiated by the National Primary Health Care Development Agency to address the acute shortage of skilled birth attendants in rural areas.

### Data source

The data for this study was extracted from the individual women recode data file for the 2013 Nigeria Demographic Health Survey (NDHS). NDHS 2013 is the fifth round of a nationally representative survey conducted to monitor population and reproductive health among Nigerians. Sampling and data collection techniques of the NDHS 2013 are described in the full published report [7]. The NDHS 2013 used a stratified 3-stage cluster design to select eligible respondents. The primary sampling units, referred to as clusters in this study were enumeration areas selected from a sampling frame prepared for the 2006 population and housing census. With a fixed sample of 45 households per 904 clusters (rural – 532; urban – 372), a total of 40, 680 households were selected and 38, 948 women aged 15–49 years successfully interviewed. Analysis was restricted to women with an index birth within 5 years preceeding the survey.

### Variables

The dependent variable in this study was dropout from maternity care continuum which was considered in 3 stages. Stage 1(model I) is the level of antenatal care (ANC) at which dropout was coded ‘1’ for those who did not receive antenatal care and ‘0’ for those who did. ANC here means at least one visit with a doctor, nurse or midwife providing care. In stage 2 (model II), those who got antenatal care but did not received skilled delivery assistance (from doctor, nurse/midwife) were deemed to be dropout and coded 1 (and otherwise 0). At the third stage (model III), those who got skilled delivery assistance but without the 6 week postnatal care were deemed to have dropped out and subsequently coded 1 (and otherwise 0).

Antenatal care was derived from response to questions 408 and 409 asking women concerning their last birth within 5 years before the NDHS 2013. The questions were “*(1) did you see anyone for antenatal care for this pregnancy*?” [Yes/NO]. Those who answered in the affirmative were further asked “*whom did you see?*” For this study, those who saw a doctor, nurse/midwife were classified as having received antenatal care. Skilled delivery was ascertained from responses to question 433 which was “*who assisted with the delivery of (NAME)*?” Those that were assisted by doctor, nurse/midwife were deemed to have received skilled delivery assistance. Status of sixth week postnatal checkup was derived from question 442 (“*In the two months after (NAME) was born, did any healthcare provider check on his/her health.....”*). Women who gave a ‘yes’ response to the question were categorized as having received the 6th week postnatal checkup.

For each model, we controlled for other covariates which are known to be associated with maternal healthcare utilisation [8, 9]. These included: maternal age, birth order, education, household wealth status, partner’s education, decision making authority (own health and visits to friends/families), rural/urban residence,geo-political zone and problems in accessing health care (getting permission to go to health facility, getting money for treatment and consultation, distance to health facility, fear of going alone, and attitude of health workers).

### Statistical modelling

We fitted mutilevel logistic regression models for each stage of dropout from the maternity care continuum described above to identify the associated independent factors. The multilevel model serves 2 purposes. First, it enabled us to control for dependence in data collected among respondents who live in the same neighbourhood (clusters). Secondly, the multilevel model allowed us to control for the effect of unmeasured/unobserved or latent variables at the cluster (community) level. Intentionally, no contextual variables were derived for inclusion in the analyses because investigation of contextual variables was not our primary interest in this study. Measure of fixed effects were expressed as Odds Ratio (OR) with their 95 % conficence interval (CI).

### The models were of the form

$$ {Y}_{ij}={0}_j+{k}_j\left({X}_{ikj}\right)+{e}_{ij} $$

Where:

*Y*_*ij*_ = log odds of dropout at any stage of the maternity care continuum for woman i in cluster (community) j

*0*_j_ = intercept for individual-level model (average risk of dropout at any stage in cluster j)

*X*_*ikj*_ = covariates (education, age group, wealth index, etc)

*K*_*j*_ = coefficients for the individual level covariates

*e*_*ij*_ = error terms for the individual-level model

We estimated the intra-cluster correlation (ICC) in the dependent variable for stages I to III. The likelihood ratio test was used to check the significance of the random effects (intra-community correlation). The ICC capture the proportion of the total variation in risk of dropout that is attributable to differences between cluster.

## Results

### Subject characteristics

About 3 quarter (75.6 %) of the study subjects were aged 18–34 years; 18.5 % were in age group 35 years and above. Distribution of the birth order shows that 50.6 % of the index birth were of order 4 and above. Close to half of the women (47.9 %) had no formal education while only 6.3 % attained tertiary education. The commonest occupation was sales/clerical work (39.5 %) and the least were professionals/technical/managerial/services (8.4 %). Majority of the women belonged to the poor wealth quintile (45.4 %). Partner’s education had a similar distribution as that of the women, the highest proportion being those with no formal education (39.9 %) . In terms of regional distribution, the North west (36.4 %) had the largest proportion of women followed by the North East (16.8 %). The women were asked whether certain issues constituted a problem for them in accessing healthcare. The responses are presented in the first few rows of Table 1. The most common problems reported were difficulty in getting money for treatment and consultation (43.8 %) and distance to health facility (31.6 %).

**Table 1:** Respondents’ characteristics

Variables	Total No of women (*n* = 20, 467)	% of all women
Problems in accessing healthcare		
Getting permission to go	2501	12.2
Getting money	8956	43.8
distance to health facility	6472	31.6
Not wanting to go alone	2934	14.3
Attitude of health workers	3434	16.8
Maternal age at child birth (Years)		
< 18	1212	5.9
18 - 34	15478	75.6
> = 35	3778	18.5
Birth order		
1	3671	17.9
2	3361	16.4
3	3054	14.9
4+	10382	50.7
Highest Education attained		
None	9795	47.9
Primary	3914	19.1
Secondary	5475	26.7
Higher	1283	6.3
Occupation		
Not working	6056	29.6
Professional/technical/managerial/services	1713	8.4
Sales/clerical	8092	39.5
Manual	4605	22.5
Wealth index		
Poor	9286	45.4
Middle	3902	19.1
Rich	7279	35.6
Partners’ education		
None	7981	39.9
Primary	3661	18.3
Secondary	5806	29.0
Higher	2566	12.8
Decision making on own health		
Yes	7065	34.5
No	13402	65.5
Decision making on visit to friends/relatives		
Yes	8780	42.9
No	11689	57.1
Residence		
Urban	7279	35.6
Rural	13189	64.4
Region		
North Central	2890	14.1
North East	1657	16.8
North West	7445	36.4
South East	1720	8.4
South South	2001	9.8
South West	2976	14.5

### Patterns of dropout from MNCH continuum

The proportion of women who received antenatal care, delivery by skilled birth attendants and postnatal care were 60.6 %, 37.5 % and 27.9 % respectively. Table 2 shows the pattern of dropouts from maternity care continuum according to subject characteristics. Among 12,392 women who received antenatal care, 38.1 % dropped out and never got skilled delivery care. Of those who received skilled delivery care, 50.8 % did not attend postnatal visit.

**Table 2:** Pattern of dropout from maternity care continuum in Nigeria

Variables	Number of women (n)			% of women		
	All women	Had ANC but No Delivery care	Had delivery care but no 6 weeks PNC	No ANC	Had ANC but No Delivery care	Had delivery care but no 6 weeks PNC
Problems in accessing healthcare						
Getting permission to	2501	821	431	67.2	55.8	65.4
Getting money	8956	4552	2913	49.2	43.5	59.8
Distance to health facility	6472	2661	1467	58.9	52.0	65.1
Can’t go alone	2934	1005	558	65.7	53.4	67.1
Attitude of health workers	3434	1690	977	50.8	46.0	60.9
Maternal age at child birth (Years)						
< 18	1212	495	280	59.2	51.7	55.4
18 - 34	15478	9594	6244	38.0	37.2	50.5
> = 35	3778	2306	1566	39.0	38.6	51.1
Birth order						
1	3671	2440	1866	33.5	27.5	49.9
2	3361	2183	1506	35.0	32.7	47.6
3	3054	1935	1306	36.6	35.2	48.2
4+	10382	5837	3412	43.8	45.4	53.6
Highest Education attained						
None	9795	3549	1200	63.8	71.3	62.7
Primary	3914	2800	1779	28.5	40.6	54.5
Secondary	5475	4796	3859	12.4	20.5	49.0
Higher	1283	1249	1252	2.7	5.2	39.6
Occupation						
Not working	6056	3034	1802	49.9	45.5	55.2
Professional/technical/managerial/services	1713	3399	1362	12.2	14.3	45.3
Sales/clerical	8092	5104	3217	36.9	36.6	47.6
Manual	4605	2752	1709	40.2	45.6	56.6
Wealth index						
Poor	9286	3212	1152	65.4	71.7	67.7
Middle	3902	2645	1657	32.3	44.7	55.8
Rich	7279	6537	5281	10.2	18.9	45.5
Partners’ education						
None	7981	2634	898	67.0	71.8	62.2
Primary	3661	2463	1524	32.8	41.5	57.4
Secondary	5806	4650	3437	19.9	26.7	49.5
Higher	2566	2291	1965	10.7	20.0	42.3
Decision-making on own health						
Yes	7065	5360	4266	24.1	21.8	48.2
Decision-making on visit to friends/relatives						
Yes	8780	6404	4969	27.1	23.8	48.0
Residence						
Urban	7279	6258	4741	14.0	22.5	46.7
Rural	13189	6135	3349	53.5	54.0	56.5
Region						
North Central	2890	1937	1639	33.0	36.6	50.0
North East	1657	1692	859	50.7	61.9	50.3
North West	7445	3053	761	59.0	70.2	56.0
South East	1720	1557	1410	9.4	11.0	64.8
South South	2001	1461	1234	27.0	26.5	56.6
South West	2976	2692	2187	9.6	9.5	37.4
Nigeria	20467	12392	8090	39.4	38.1	50.8

The most common problems that hindered women from accessing antenatal care was inability to get permission (67.2 %) and inability to go alone (65.7 %). Women younger than 18 years (59.2 %) had the greatest likelihood of not accessing antenatal care. Similarly, non-use of antenatal care increased with birth order from 33.5 % among first birth women to 43.8 % in those with 4 or more living children. In terms of educational distribution, 63.8 % of women with no formal and 2.7 % of those who attained higher education did not accessed antenatal care. Women in rural areas (53.5 %), North East (50.7 %) and North West (59.0 %) constituted the highest percentage without antenatal care.

The distribution of dropout between antenatal and delivery is presented in the fifth column of Table 2. The factors associated are similar to those related to non-use of antenatal care. For instance, dropout at this stage was more common among women less than 18 years (51.7 %) and those with birth order 4 and above (45.4 %). The percentage of women who dropped out between ANC and delivery among those without formal education and primary level education was 71.3 % and 40.6 % respectively. Similarly, women in the poor wealth quintile recorded higher (71.7 %) dropout than middle (44.7 %) and rich (18.9 %) quintile. Across regions, the Southwest (9.5 %) and South East (11.0 %) had the lowest level of dropout between ANC and delivery.

Substantial dropout was recorded between delivery and the sixth week postnatal checkup. At this point, differentials in dropouts were also noticeable across most variables. About 65 % of women who had problem with getting permission to go for healthcare or distance to health facility defaulted from the sixth week postnatal care. Slightly more than half of women aged less than 18 years (55.4 %) and 53.6 % of those with births order of 4 and above also dropped out. Women without formal education (62.7 %) and those in the poor wealth quintile (67.7 %) had the greatest incidence of dropout. The percentage of dropout varied across regions with values ranging from 37.4 % in the South west, 50.9 % in the Northwest to 64.8 % in the South East.

### Correlates of dropout from maternity care continuum

Results of the multilevel logistic regression model fitted to identify the independent factors associated with dropouts from maternity care continuum are presented in Table 3. Model I shows that some problems in accessing healthcare, birth order, education, occupation, wealth quintile, partner’s education, place of residence, and region are predictors of non-use of antenatal care services. Of the healthcare access problems, difficulty in getting permission (OR = 1.74, CI:1.51–2.02) had the strongest association with non-use of antenatal care. The odds of non-use of ANC increased with birth order. Women without formal education were 6 times as likely as those with higher education not to attend ANC (OR = 5.98, CI: 4.11–8.72). Respondents in rural areas were almost 3 times more likely not to access ANC compared to their urban counterparts (OR = 2.58; CI: 2.05–3.25). Women in the North East (OR = 1.97; CI: 1.35–2.88), North West (OR = 3.64; CI: 2.54–5.23) and South South (OR = 3.62; CI: 2.51–5.24) regions had the greatest likelihood of not utilising antenatal care. The random effect shows an ICC of 30.89 % which is indicative of the proportion of the variation in non-use of ANC that is due to unmeasured/unobserved charcatersitics shared by women from the same communities.

**Table 3:** Determinants of dropout from maternity care continuum in Nigeria

Variables	Model 1:No ANC	Model II: Had ANC but No delivery care	Model III: delivery care but No PNC
Problems in accessing healthcare	OR (95 % CI)^a^	OR (95 % CI)^a^	OR (95 % CI)^a^
Getting permission to go	1.74 (1.51-2.02)*	0.84 (0.67-1.05)	1.14 (0.88-1.47)
Getting money	1.19 (1.08-1.31)*	1.18 (1.04-1.34)*	1.18 (1.04-1.35)*
Distance to health facility	1.22 (1.09-1.36)*	1.31 (1.13-1.52)*	1.32 (1.12-1.57)*
Can't go alone	1.37 (1.19-1.58)*	1.07 (0.86-1.33)	1.16 (0.89-1.48)
Attitude of health workers	0.95 (0.84-1.09)	0.83 (0.69-0.99)	1.14 (0.95-1.38)
Maternal age at child birth (yrs)			
< 18	1.00	1.00	1.00
18 - 34	1.25 (0.99-1.58)	1.28 (0.93-1.75)	1.10 (0.76-1.59)
> = 35	1.01 (0.90-1.13)	1.28 (1.11-1.48)*	1.09 (0.94-1.26)
Birth order			
1	1.00	1.00	1.00
2	1.15 (0.99-1.35)	1.67 (1.39-2.02)*	0.97 (0.82-1.15)
3	1.25 (1.06-1.47)*	4.22 (3.05-5.83)*	1.01 (0.84-1.20)
4+	1.36 (1.18-1.58)*	2.84 (2.08-3.86)*	1.17 (0.97-1.39)
Highest Education attained			
None	5.98 (4.11-8.72)*	5.19 (3.71-7.25)*	1.78 (1.37-2.32)*
Primary	3.66 (2.53-5.30)*	4.22 (3.05-5.83)*	1.29 (1.04-1.62)*
Secondary	2.28 (1.59-3.26)*	2.84 (2.08-3.86)*	1.17 (0.97-1.39)
Higher	1.00	1.00	1.00
Occupation			
Not working	1.00	1.00	1.00
Professional/technical/managerial/services	0.73 (0.59-0.90)*	0.91 (0.73-1.44)	0.87 (0.73-1.04)
Sales/clerical	0.71 (0.64-0.80)*	0.96 (0.83-1.11)	0.79 (0.68-0.92)*
Manual	0.75 (0.67-0.85)*	1.07 (0.91-1.25)	0.93 (0.78-1.11)
Wealth index			
Poor	2.57 (2.17-3.05)*	2.22 (1.85-2.67)*	1.65 (1.32-2.07)*
Middle	1.59 (1.37-1.84)*	1.61 (1.38-1.87)*	1.15 (0.98-1.36)
Rich	1.00	1.00	1.00
Partners’ education			
None	2.30 (1.91-2.77)*	1.86 (1.52-2.27)*	1.14 (0.90-1.45)
Primary	1.50 (1.24-1.81)*	1.56 (1.29-1.89)*	1.12 (0.93-1.35)
Secondary	1.48 (1.24-1.76)*	1.30 (1.10-1.53)*	1.07 (0.92-1.24)
Higher	1.00		1.00
Decision making on own health			
Yes	0.98 (0.86-1.12)	0.88 (0.75-1.03)	0.94 (0.81-1.09)
Decision making on visit to friends/relatives			
Yes	0.94 (0.83-1.06)	0.91 (0.77-1.06)	1.03 (0.88-1.19)
Residence			
Urban	1.00	1.00	1.00
Rural	2.58 (2.05-3.25)*	1.98 (1.63-2.41)*	1.12 (0.92-1.37)
Region			
North Central	1.38 (0.96-1.99)	2.79 (2.07-3.77)	1.54 (1.16-2.04)*
North East	1.97 (1.35-2.88)*	8.49 (6.12-11.79)*	1.26 (0.89-1.77)
North West	3.64 (2.54-5.23)*	15.11 (10.97-20.82)*	1.84 (1.31-2.60)*
South East	0.57 (0.37-0.87)*	0.66 (0.46-0.95)*	3.12 (2.32-4.20)*
South South	3.62 (2.51-5.24)*	3.41 (2.49-4.67)*	1.89 (1.40-2.55)*
South West	1.00	1.00	1.00
Random effects: community level			
Variance (SE)	1.2125 (0.0452)*	0.9014 (0.0473)*	0.9873 (0.0469)
ICC (%)	30.89 %	19.81 %	22.86 %

*p < 0.05 (*p*-values based on Wald test); ^**a**^Odds Ratio adjusted for all other variables in the table

From model II, we identified the predictors of dropout between antenatal care and delivery among women who received ANC. Getting money to go (OR = 1.18, CI: 1.04–1.34) and distance to health facility (OR = 1.31, CI: 1.13–1.52) were the significant healthcare access problems. The odds of dropout at this stage was 28 % higher among women aged 35 years and above (OR = 1.28, CI: 1.11–1.48). The odds increased with birth order but decreased as educational level increases. Women in the poor (OR = 2.22, CI: 1.85–2.67) and middle (OR = 1.61, CI: 1.38–1.87) wealth qunitiles were more likely to drop out compared to those in the rich quintile. Rural residents were 2 times as likely as their urban counterparts to dropout between ANC and delivery (OR = 1.98, CI: 1.63–2.41). Regional differences between North East, North West and South West widened from model I to model II. For instance, women in North west were 15 times as likely as their South west folks to dropout (OR = 15.11, CI: 10.97–20.82) while those from the South East (OR = 0.66, CI: 0.46–0.95) were less likely. The cluster effect was still statistically significant even though the ICC (19.81 %) was smaller than that of model I.

The correlates of dropout between delivery and the 6 week postnatal check up were determined using model III. Getting money for consultation and treatment (OR = 1.18 CI: 1.04–1.35) and distance to health facility were the significant healthcare access problems at this stage. Other significant predictors were lack of formal education (OR = 1.78, CI: 1.37–2.32), primary education (OR = 1.29, CI: 1.04–1.62) and poor wealth quintile (OR = 1.65, CI: 1.32–2.07). Signifcant regional differentials were also recorded between North central (OR = 1.54, CI: 1.16–2.04), North west (OR = 1.84, CI: 1.31–2.60), South east (OR = 3.12, CI: 2.32–4.20), South south (OR = 1.89, CI: 1.40–2.55) and the South west region. Random effect due to unobserved variables was also significant with an ICC of 22.86 %.

### Reasons for dropout between antenatal care and delivery

Among women who received antenatal care but no skilled assitance at delivery (because they deliver at home or elsewhere), the 3 commonest reasons cited were (Table 4): sudden delivery/no time to get to hospital (38.1 %), it is not necessary (33.6 %) and health facility too far/no transportation (15.1 %). The results were disaggregated by the main determinants found for dropout at this stage as earlier reported in Table 3. Major variations in the stated reasons were noticeable across educational level and region. For instance, 36.2 % and 56.6 % of women with no education and higher education respectively mentioned sudden delivery as reason for default while 16.8 % (no formal education) and 8.5 % (higher education) cited lack of transportation. The excuse of sudden delivery ranged from 25.0 % in the South south to 54.1 % in the North East. Complaints about cost being too much was highest in South south (29.%) and lowest in North west (4.0 %). See Table 4 for further details.

**Table 4:** Reasons for not delivering in a health facility among women who received antenatal care, Nigeria, 2013

Variables	Cost too high	Facility not opened	Too far / No transport	Poor service	No female provider	Partner/family didn’t allow	Not necessary	Not customary	Sudden delivery	Attitude of personnel
Maternal age at child birth (Years)										
< 18	9.4	2.0	18.7	0.9	1.0	8.6	30.4	8.5	40.2	0.1
18 - 34	9.0	2.3	14.8	1.5	0.5	8.2	33.1	9.0	38.3	0.2
> = 35	9.3	2.2	14.9	1.4	0.3	6.0	36.8	9.5	36.4	0.3
Birth order										
1	9.8	2.9	16.3	1.2	0.7	8.5	29.9	7.0	39.1	0.2
2	9.6	1.5	14.3	1.8	0.4	8.7	32.6	7.6	38.8	0.3
3	8.7	2.5	14.4	1.4	0.5	9.7	31.2	9.0	39.9	0.2
4+	8.9	2.2	15.2	1.4	0.5	7.0	35.4	10.0	37.2	0.2
Highest Education attained										
None	7.2	2.0	16.8	0.6	0.5	9.4	36.2	11.3	36.2	0.0
Primary	13.6	2.6	13.1	2.3	0.5	5.1	29.9	4.9	40.8	0.3
Secondary	13.1	3.0	10.0	3.9	0.7	4.2	26.7	4.0	42.8	1.0
Higher	2.8	3.8	8.5	2.8	0.0	4.7	20.8	4.7	56.6	0.0
Wealth index										
Poor	8.5	2.4	18.6	0.7	0.6	9.2	35.6	11.0	34.8	0.0
Middle	10.1	1.8	10.4	1.3	0.3	5.7	30.7	5.8	43.9	0.2
Rich	10.0	2.3	7.5	4.1	0.4	5.3	29.4	5.7	44.1	0.9
Residence										
Urban	8.8	1.1	8.0	3.1	0.4	5.7	28.6	6.5	47.0	0.6
Rural	9.2	2.5	17.0	1.0	0.6	8.4	34.9	9.8	35.8	0.1
Region										
North Central	12.0	1.0	15.8	0.3	0.1	2.7	31.7	2.1	46.6	0.0
North East	10.4	4.9	21.8	0.9	0.4	6.5	20.6	4.7	54.1	0.1
North West	4.0	0.6	11.1	0.5	0.5	9.9	43.2	13.9	31.7	0.0
South East	17.1	2.8	19.9	3.9	1.1	4.8	16.3	2.8	39.6	0.0
South South	29.0	4.6	15.4	4.0	1.0	1.6	23.5	2.7	25.0	1.5
South West	13.0	6.7	22.0	9.1	1.0	15.9	21.8	8.6	34.8	1.1
Nigeria	9.1	2.2	15.1	1.4	0.5	7.9	33.6	9.1	38.1	0.2
sample size	1163	286	1931	180	67	1002	4290	1158	4867	27

## Discussion

The study found heavy dropouts at different stages of the maternity care continuum in Nigeria. A pregnant woman who did not access antenatal care missed opportunities for early detection and prompt treatment of complications, immunisation and micronutrient supplementation, birth preparedness and complication readiness, disease prevention through health messages and counselling [3]. Failure to access skilled assistance during delivery implied that complications may not be properly managed and hygiene practices may be compromised. This may put the mother and newborn at higher risks of infections. The postnatal period is critical for women as they may develop life threatening complications which can be promptly treated if postnatal care is accessed. Besides, postnatal care provides opportunity to emphasize the adoption of healthy behaviours for mother and child [3]. The magnitude of dropout from the care continuum in Nigeria may explain, why it appears that proven MCH interventions are not making the expected impacts.

Dropout was found to be associated with problems in accessing health care such as distance to health facility and difficulty in getting money for treatment and consultation which was recurrent at all stages in the maternity care continuum. This finding is consistent with evidence on reasons for non-use of antenatal care in a recent Nigeria study [13]. This is reflective of the harsh economic condition especially in the rural areas. Though some states in Nigeria provide free health care services for pregnant women and under-5 children, but many of these so called free programs merely waive the consultation fees. Users of the services most often do not have option than to buy essential drugs on their own as these drugs are often not available in the facilities. Another twist to this has to do with the people’s perception and level of awareness. Atimes, consultation is free and treatment are also provided free yet, pregnant women stay away because they think they are always required to pay.

The results showed that almost half of those who received antenatal care do not get skilled assitance at delivery. This is indicative of the place of delivery being other than health facility because the NDHS 2013 report actually revealed that the percentage of pregnant women who delivered in a health facility (36 %) was almost the same as those who got skilled attendance during delivery (38 %). The question then is why do pregnant women do not deliver where they can get skilled assistance? Our results showed that the most common excuse was sudden delivery/lack of time to get to health facility. Surprisingly, the prevailing reasons also varies across geo-political regions. In the North central and North east, sudden delivery was the most common excuse. For North west, the reported excuse was that it was not necessary to deliver in a health facility while sudden delivery was also the main reason in South East and South west. High cost was the leading excuse among women in the South south. The problem of sudden delivery was even the most critical reasons among those with higher education. To overcome this untenable excuse, more effort would need to be devoted to awareness about birth preparedness across all geo-political regions. Attitude to facility delivery would need to be addressed in the Northern regions. The problem of high cost in the South south deserve further research because quite a number of states in the region claimed to offer free maternal and child health care services.

Besides the reasons stated, evidence from other studies showed that some women have preference for traditional birth attendants [14, 15] or delivery at home [16]. To take advantage of this perception among the women, it may be worthwhile to develop synergy between trained midwives and traditional birth attendants. Some other studies suggested that the frequency and quality of antenatal care recieved determine whether women remain in the care continuum or not [10, 17]. Considering the deep socio-cultural affinity to traditional birth attendants, mass mobilisation through community gatekeepers and stakeholders may achieve better results. This however does not imply that the quality of maternity care should be compromised. Infact, the community women can be mobilised, enlightened and encouraged to demand for quality care whenever they visit health facilities.

This study re-affirmed the effects of socio-economic variables such as education and wealth quintile [12]. Women with no formal education and those in the poor wealth qunitile are the most likely to drop out from the care continuum. Programmes targetted at increased education of the girl child provide long-term solution. In the short term, For faster progress in maternal and child health improvement, programs should be designed such that poor and uneducated women can have access without restriction. Such programs would be more effective if they are designed to be community-directed. There are a number of pilot interventions especially in Northern Nigeria which has demonstrated the potential to make a positive difference in the rural areas [18–20]. The approach would be to ascertain the reasons why women dropout and ask their views about the possible solutions. Feasible solutions and modalities for implementation can be agreed with the community which would also be requested to nominate volunteers to be part of the implementation process.

Despite adjustment for background charcteristics and unobserved community variables, regional differentials in dropout remained statistically significant. Even in previous studies where controls for community or contextual variables have been introduced in the model, there remained significant regional differences [9, 21]. In addition, the random effects in our results were also significant in all the models fitted. These results suggest that there are other factors at work which have not been controlled in the models. This underscore the need for further qualititative studies on the perceived or actual barriers to continuity of maternity care.

A closer look at the regional differences in dropout pattern showed that it varies from one stage to another. Between antenatal care and delivery, women in the South East were found less likely to drop out compared to those from the South west but this was reversed between delivery and postnatal care. The reversal showed that South East women were more likely to dropout after delivery. A further study on the extent of use of 6 week postnatal care among South east women could reveal the factors and mechanisms responsible for this. The North east, North west and South south regions had the greatest dropouts when compared to the South West despite adjustment for background characteristics which have often been cited as reasons for regional differences. It is suspected that difficulty with transportation may underlie these patterns. In the North east and North west, lack of transportation from remote rural areas to health facility has been found to be a huge challenge [18]. In the South south, several communities are in the riverine areas where transportation is limited. Pregnant women or women with newborns may not be willing to go through the risk and stress of water transportation. To circumvent this problem of transportation and distance to health facility, mobile health care services have been tried in the North west [19]. However, this may not be adequate for the maternity care continuum. Rather, a community-based approach in which community health extension workers who live in the rural areas are trained to provide care may be more effective. Other programmes with promise to improve retention in maternity care include tracking of women through mobile phone/sms reminders, conditional cash transfers, home-based postnatal care (especially for home deliveries) and community-driven initiatives [12].

There are some limitations in this study. We could not ascertain the reasons for dropout between delivery and sixth week postnatal care because such data were not collected in the NDHS 2013. Being a cross-sectional data, causality could be ascertained but relationships have been established. A strength of this study lies in the currency and nationally representativeness of the data to inform programs and policies aimed at strengthening the maternal care continuum in Nigeria. Another plus for this study is the approach taken to investigate continuity of care unlike most previous studies that addressed utilisation of individual maternal healthcare services.

## Conclusion

The rate of dropout from maternity care continuum is high in Nigeria and driven by low or lack of formal education, poverty, and socio-economic problems (distance to facility and difficulty with getting money for treatment). Unexpectedly, dropouts are high in South east and South south as well as the Northern regions. Further studies are needed to fully understand why women do not complete the maternity care continuum. Besides, programs promoting maternal health utilisation should emphasize accessing the full package rather than only specific components. Innovative advocacy programs focusing on community outreach about the continuum of maternal healthcare package should be introduced especially for women in rural areas and lower socio-economic strata.

## Abbreviations

ANC: Antenatal Care
CI: Confidence Interval
ICC: Intra-cluster correlation
MCH: Maternal and Child Health
MDG: Millennium Development Goals
MNCH: Maternal Newborn and Child Health
MSS: Midwives Service Scheme
NDHS: Nigeria Demographic and Health Survey
OR: Odds Ratio
SDG: Sustainable Development Goals
